# Differential response of bacterial diversity and community composition to different tree ages of pomelo under red and paddy soils

**DOI:** 10.3389/fmicb.2022.958788

**Published:** 2022-07-22

**Authors:** Chaoyuan Zheng, Kunpeng Kong, Yi Zhang, Wenhao Yang, Liangquan Wu, Muhammad Zeeshan Munir, Baoming Ji, Muhammad Atif Muneer

**Affiliations:** ^1^College of Resources and Environment/International Magnesium Institute, Fujian Agriculture and Forestry University, Fuzhou, China; ^2^School of Environment and Energy, Peking University Shenzhen Graduate School, Shenzhen, China; ^3^College of Grassland Science, Beijing Forestry University, Beijing, China

**Keywords:** pomelo orchard, tree ages, bacterial community, environmental factors, red and paddy soils

## Abstract

Rhizosphere soil microbial communities substantially impact plant growth by regulating the nutrient cycle. However, dynamic changes in soil microbiota under different tree ages have received little attention. In this study, changes in soil physicochemical properties, as well as bacterial diversity and community structures (by high-throughput Illumina MiSeq sequencing), were explored in pomelo trees of different ages (i.e., 10, 20, and 30 years) under red and paddy soils cultivated by farmers with high fertilizer input. Moreover, soil factors that shape the bacterial community, such as soil pH, AP (available phosphorous), AK (available potassium), and AN (available nitrogen), were also investigated. Results showed that pH significantly decreased, while AP, AK, and AN increased with increasing tree age under red soil. For paddy soil, pH was not changed, while AP was significantly lower under 10-year-old pomelo trees, and AK and AN contents were minimum under 30-year-old pomelo trees. Both soil types were dominated by *Proteobacteria, Acidobacteria*, and *Actinobacteria* and showed contrasting patterns of relative abundance under different tree age groups. Bacterial richness and diversity decreased with increasing tree age in both soil types. Overall, bacterial community composition was different under different tree ages. RDA analysis showed that soil pH, AP, and AN in red soil, and pH and AP in paddy soil showed the most significant effects in changing the bacterial community structure. A random forest model showed *Sinomonas* and *Streptacidiphilus* in red soil, while *Actinoallomurus* and *Microbacterium* in paddy soil were the most important genera explaining the differences among different age groups. The ternary plot further revealed that genera enrichment for Age_30 was higher than that for Age_10 and Age_20 in red soil, whereas specific genera enrichment decreased with increasing tree age under paddy soil. Co-occurrence network revealed that bacterial species formed a complex network structure with increasing tree age, indicating a more stable microbial association under 20 and 30 years than 10-year-old pomelo trees. Hence, contrasting patterns of changes in soil physicochemical properties and soil microbial communities were recorded under different tree ages, and tree ages significantly affected the bacterial community structure and richness. These findings provide valuable information regarding the importance of microbes for the sustainable management of pomelo orchards by optimizing fertilizer input for different ages of trees.

## Introduction

Soil microbes, especially bacteria, which represent the most abundant group accounting for ≈80% of total microbiota in the terrestrial ecosystem (Gans et al., 2006), play central roles in various biochemical and ecological processes, including carbon and nitrogen cycles and decomposition of organic matter (Gougoulias et al., 2014; Diao et al., 2020). Various factors, like soil pH, available phosphorous (AP), and carbon-to-nitrogen ratio (C:N ratio), may influence soil bacterial diversity (Li et al., 2021; Sheng et al., 2021). It is generally known that tree age and species can affect soil microbial diversity and community composition directly or indirectly by changing the circulation of soil nutrients (Sun et al., 2014; Du et al., 2018). The variation in soil microbial population reflects the dynamics of nutrient cycling and soil health. Hence, it is imperative to figure out how the bacterial population changes in response to varying tree ages.

Previous studies have shown that soil microbial communities vary under different stand ages. For example, *Robinia pseudoacacia* and *Pinus tabuliformis* have distinctive soil bacterial populations (Dang et al., 2017; Liu et al., 2018). These differences in bacterial community and diversity could result from an increase in the tree canopy density with increasing tree age, which in turn reduces the light availability and increases the soil moisture content, leading to structural changes of the microbial community (Qu et al., 2020). So far, various studies have examined the changes in bacterial diversity and community composition across different tree ages in the forest tree species (Wu et al., 2015; Zhou et al., 2017; Zhu et al., 2019). However, few studies have explicitly focused on how the host age could affect the soil microbial diversity and community composition of the orchards, especially in the citrus orchard.

Citrus, an evergreen fruit tree grown in tropical and subtropical regions, is the leading fruit crop globally and contributes to many countries' economies and fruit production (Liu et al., 2012). China is among the world's top citrus producers, with an annual production of 4,406 × 10^4^ tons (Huang et al., 2021). Pomelo (*Citrus grandis*) is China's third most popular citrus type after *Citrus reticulata* and *Citrus sinensis* (Li et al., 2015). Hence, the land-use change results in diverse management practices, including fertilization, cultivation methods, and irrigation, which significantly affect the soil quality and may lead to changes in soil microbial diversity and community composition (Guo et al., 2019). Therefore, understanding the underlying differences in microbial communities in the red and paddy soils is of great interest. Moreover, it has also been documented that as the stand age increases, soil nutrient status is also changed, and as a result, soil microbial communities change, including forest and orchard systems (Kyaschenko et al., 2017; Zhang et al., 2017; Antisari et al., 2018; Wu et al., 2020). Citrus belongs to perennial tree species that frequently undergo progressive growth and changes year by year, and plant age substantially affects rhizosphere bacterial composition (Ji et al., 2019). Many earlier research studies have emphasized the effects of planting on soil physicochemical properties. By contrast, little attention has been paid to how the changes in planting age could affect the soil microbial communities.

In this study, we used high-throughput Illumina MiSeq sequencing technology to investigate the bacterial community structures in pomelo trees of different ages under the red and paddy soils. This study aimed to determine (a) bacterial diversity and community composition under pomelo trees of different ages, (b) the environmental factors influence the bacterial community composition, and (c) the key species of bacterial communities residing in the red and paddy soils. These research findings could lead to future sustainable management of pomelo trees.

## Materials and methods

### Sampling site and collection of soil samples

This study was conducted in Pinghe County (24°02′-24°35′N, 116°54′-117°31′E), Fujian Province, southern China, which belongs to a subtropical monsoon climate with an annual average temperature of 25.38°C and humidity 1,600 mm (Yan X. et al., 2021). The soil samples were collected from red and paddy soils of the pomelo orchards of Pinghe County. These soil samples were collected from pomelo trees of different ages, that is, 10 years (Age_10), 20 years (Age_20), and 30 years (Age_30). A total of 57 soil samples were collected from red soil (i.e., topsoil 0–20 cm), and 10-, 20-, and 30-year-old trees had 20, 22, and 15 samples. In the case of paddy soil, 32 soil samples were collected from topsoil, and 10-, 20-, and 30-year-old trees had 15, 11, and six samples because of the limited availability of trees in the selected groups of ages. Soil samples were transported to the laboratory on ice. Soil samples were sieved through a 2-mm-diameter mesh, and the straw residues and fine roots were removed manually. The soil samples were divided into parts for determining soil properties (stored at 4°C) and molecular analysis (stored at −80°C).

### Determination of soil physicochemical properties

In order to determine soil pH, we prepared a soil/water suspension in the ratio of 1:2.5 (*w/v*), and it was measured by using a pH meter (ORION A215STAR, Thermo Ltd., USA) (Zhang et al., 2021). Available nitrogen (AN) was determined by sodium hydroxide (NaOH) hydrolysis. In short, soil was treated with NaOH, hydrolyzed-N was converted into NH_3_, and then absorbed by H_3_BO_3_. At the final stage, hydrolyzed-N was titrated with H_2_SO_4_ (Zhou et al., 2022). Available phosphorous (AP) was extracted using 0.5 mol L^−1^ of sodium bicarbonate (NaHCO_3_) at pH 8.5 and quantified by a SpectraMax M4 spectrophotometer (Molecular Devices, Ca, USA) (Yan et al., 2022). Available potassium (AK) was extracted with ammonium acetate solution (NH_4_CH_3_CO_2_) and quantified by flame photometry (FP6410, INESA, China) (Guo et al., 2022).

### Soil DNA extraction

For total soil DNA extraction, 0.5 g of soil sample was used for each soil type of red and paddy soils by following the instructions of the soil DNA extraction kit (MO-BIO Laboratories, Carlsbad, CA-USA). To check DNA purity and concentration, agarose gel electrophoresis and a Nanodrop-2000 spectrophotometer (Thermo-Scientific) were used. An adequate sample quantity was collected in a centrifuge tube, diluted with sterile water to 10 ng/μl, and stored at −40 °C for further analysis.

### PCR analyses and high-throughput sequencing

To amplify the soil DNA, specific primers of 515F (5′-GTGCCAGCMGCCGCGGTAA-3′) and 909R (5′-CCCCGYCAATTCMTTTRAGT-3′) were used, which cover the bacterial 16S V4–V5 amplification region. PCR was performed with a 25 μl mixture containing PCR buffer (1 ×), MgCl_2_ of 1.5 mM, deoxynucleoside triphosphate of 0.4 mM, and 0.5 U TaKaRa Ex-Taq. The concentration of soil genomic DNA was 10 ng, and each primer was 1.0 μM. The PCR was performed with an initial denaturation step at 94°C for 3 min, followed by 30 cycles at 94°C for 40 s, 56°C for 60 s, and the final extension at 72°C for 10 min. After each sample's PCR amplification, the two PCR results were combined (Li et al., 2015). Gel electrophoresis containing 1% agarose was used to run both PCR products, and the targeted bands of DNA were extracted and purified with a gel extraction kit. To quantify PCR products, a NanoDrop (Thermo Scientific NanoDrop-2000) spectrophotometer was used. For sequencing, the samples were prepared according to the manufacturer's instructions of the TruSeq DNA kit. Qubit and qPCR were used to analyze the constructed library and then sequenced on the Illumina Miseq system (Bobett Biotechnology Co., Ltd., Sichuan, China).

### Sequencing data processing

The raw sequences were processed with the default parameters and UPARSE workflow using the Quantitative Insights Into Microbial Ecology (QIIME v1.9.0) tool. The low-quality reads, primers, and barcode sequences were all excluded from the analysis. At 97% sequence similarity, the operational taxonomic units (OTUs) were grouped. The sequencing data are available at the NCBI BioProject SRA database under accession number PRJNA779204.

### Data analyses

Species richness and alpha diversity were calculated based on alpha diversity indices, including Observed, Chao1, and Shannon. We used KW (Kruskal–Wallis) tests to determine the significant difference in the alpha diversity index between different tree age groups in the red and paddy soils. The variation in bacterial beta diversity among different tree ages was assessed using principal coordinate analysis (PCoA) based on Bray–Curtis dissimilarity. The permutational multivariate analysis of variance (PERMANOVA) method was also used to investigate whether bacterial populations differed significantly between different tree ages. Random forest analysis was applied to identify the most important bacterial taxa in red and paddy soils by using a random forest package. To further investigate which bacterial community significantly increased or decreased under different tree ages, ternary plot analysis was performed in R language-based package DESeq2, ggplot2, and grid to investigate the enriched and depleted bacterial community among different tree age groups. To visualize the overlapping and unique enriched genera among different age groups, we used Venn diagrams (Venny 2.1.0) (Pang et al., 2021, 2022). Based on Spearman's correlation, we explored the network of co-occurrences of bacterial communities. The OTUs were selected based on significant correlation (*P* < 0.01, ρ > 0.7). Taxonomic abundances were analyzed pairwise, resulting in an extremely complex network in which each node represents a phylum and the internodes represent significant associations. Gephi V0.9.2 was used to visualize and modularize co-occurrence. All these statistical analyses were performed using the R package “microeco v0.2.0” (Liu et al., 2020).

## Results

### Changes in soil physicochemical properties under different tree ages

**Figure 1:**
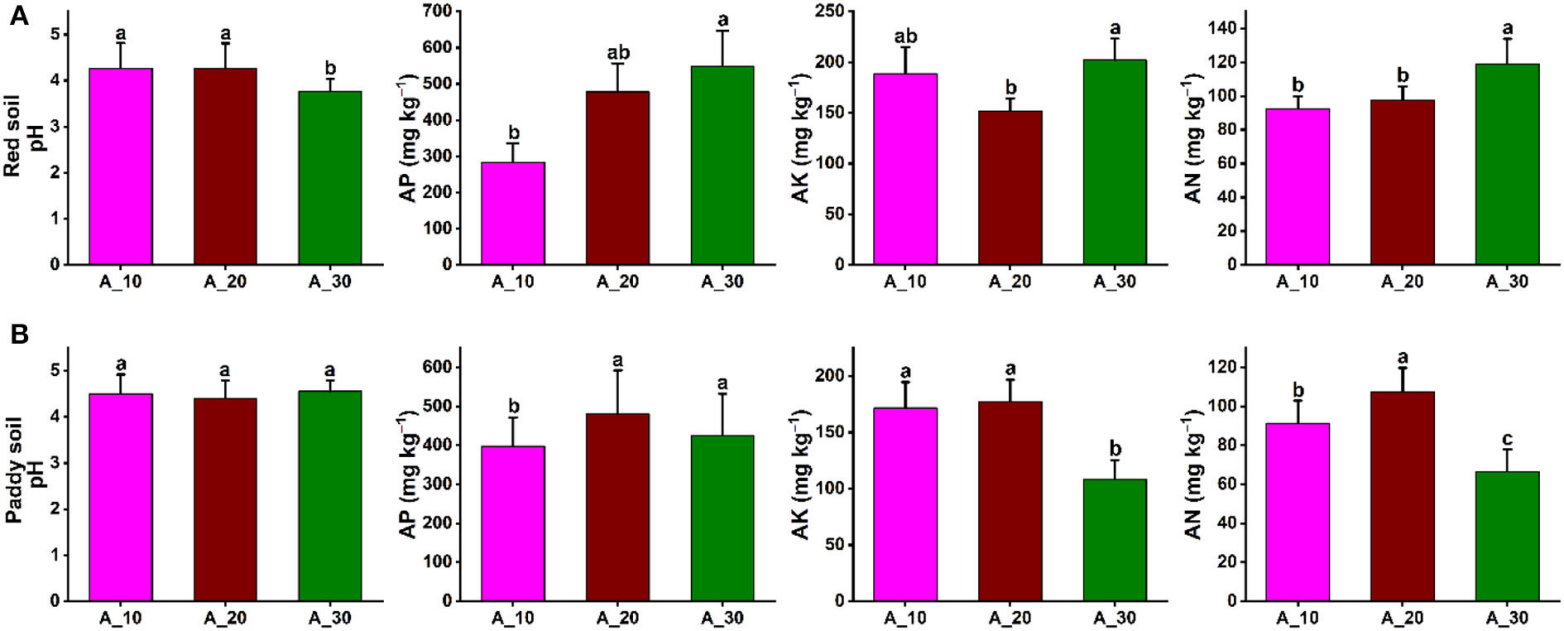
Variations in soil properties under different tree ages. Variation in soil physicochemical properties (soil pH, AP, AK, and AN) under different groups of tree ages for **(A)** red soil **(B)** paddy soil. Here, AP, AK, and AN represent available phosphorous, available potassium, and available nitrogen, respectively. Different lowercase letters indicate the significant differences (*p* < 0.05) among different pomelo trees age groups of Age_10 (10 years old), Age_20 (20 years old), and Age_30 (30 years old).

In red and paddy soils of pomelo orchards, the soil physicochemical properties were significantly affected by increasing tree age. In red soil, soil pH was significantly decreased with increasing tree age, that is, 4.26, 4.27, and 3.76 under 10-, 20-, and 30-year-old trees, respectively. By contrast, AP, AK, and AN significantly increased with increasing tree age, and maximum contents of 549, 202, and 119 mg kg^−1^ were recorded, respectively (Figure 1A). In the case of paddy soil, tree age also had a significant effect on soil physicochemical properties. The soil pH was not significantly changed with increasing tree age, and the average soil pH of 4.48 was recorded. The AP was significantly lowest for 10-year-old trees. The AK and AN contents also decreased significantly with increasing tree age, and minimum contents were observed in 30-year-old pomelo trees. AK contents were 172, 177, and 108 mg kg^−1^, while for AN, 91, 107, and 67 mg kg^−1^ contents were recorded for 10-, 20-, 30-year-old trees, respectively (Figure 1B). These results showed contrasting patterns of changes in soil physicochemical properties under different tree ages in the red and paddy soils.

### Variation in the bacterial community structure under different tree ages

**Figure 2:**
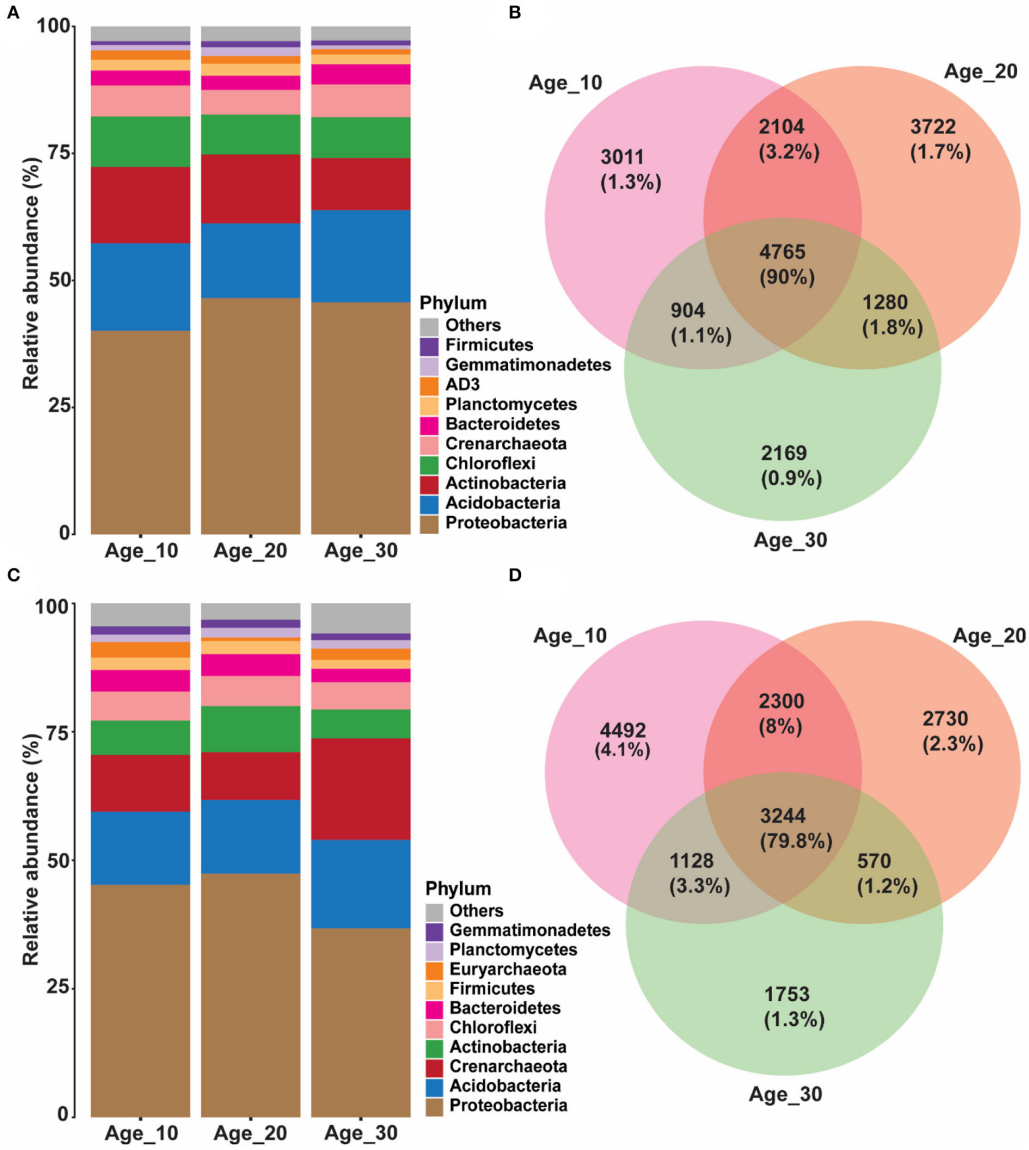
Variations in relative abundance of soil bacterial community under different tree ages. Changes in relative abundance and comparison of bacterial community were observed under different tree ages; **(A,B)** red soil; and **(C,D)** paddy soil. Relative abundance has been shown of top 10 bacterial community at phylum level. The different lowercase letters indicate the significant differences (*p* < 0.05) among different pomelo trees age groups of Age_10 (10 years old), Age_20 (20 years old), and Age_30 (30 years old).

Overall, 24,327 OTUs were obtained after removing chimeras and resampling. Red soil was dominated by bacterial profiles of *Proteobacteria* (44.03%), *Acidobacteria* (16.51%), *Actinobacteria* (13.19%), *Chloroflexi* (8.67%), *Bacteroidetes* (3.16%), *AD3* (1.50%), *Gemmatimonadetes* (1.24%), and *Firmicutes* (0.97%). In different groups of tree ages, the average relative abundance of *Proteobacteria* was highest in Age_10 (41.26%), Age_20 (47.90%), and Age_30 (46.96%) compared to other phyla. However, relative abundance of *Proteobacteria* increased with increasing tree age. The second most abundant phylum was *Acidobacteria*, with average relative abundances of 17.76, 15.14, and 18.68% in Age_10, Age_20, and Age-30, respectively. The relative abundance of *Acidobacteria* was highest in Age_30 (Figure 2A). The Venn diagram revealed 4,765 shared OTUs by different age groups, and the shared OTUs were characterized by 90% of total reads (Figure 2B).

Similarly, the paddy soil was also dominated by the bacterial communities of *Proteobacteria* (44.39%), *Acidobacteria* (14.83%), *Actinobacteria* (7.27%), *Chloroflexi* (5.65%), *Bacteroidetes* (3.93%), *Firmicutes* (2.32%), *Planctomycetes* (1.63%), and *Gemmatimonadetes* (1.52%). The average relative abundance of *Proteobacteria* was high compared to other bacterial phyla in Age_10 (45.23%), Age_20 (47.40%), and Age_30 (36.75%). Hence, in contrast to red soil, the relative abundance of *Proteobacteria* was decreased at Age_30 in paddy soil. In paddy soil, the second most abundant phylum was *Acidobacteria*, with average relative abundances of 14.22, 14.34, and 17.21% at Age_10, Age_20, and Age-30, respectively, and with the highest relative abundance under Age_30 (Figure 2C). However, an average relative abundance of *Actinobacteria* was low under paddy soil compared with red soil. The Venn diagram showed that different age groups shared 3,244 OTUs, accounting for 79.8% of total reads (Figure 2D). It showed that the similarity of the bacterial community of varying tree ages under red and paddy soils had a substantial degree of similarity, and their bacterial profiles had a generally stable composition for ecological functioning.

### Bacterial richness and diversity under different tree ages in red and paddy soils

**Figure 3:**
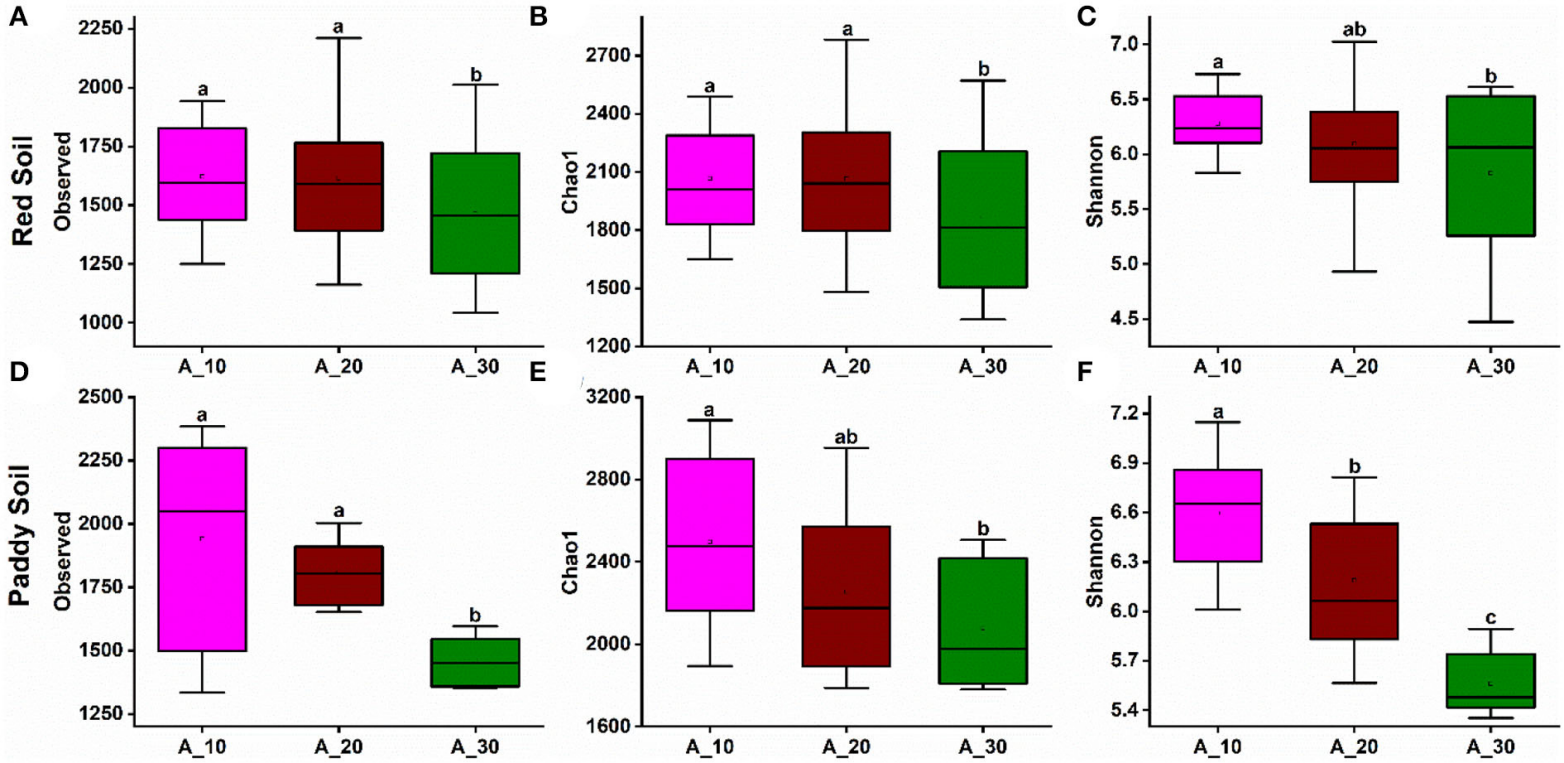
Alpha diversity indexes under different tree ages. Changes in alpha diversity indices, including observed, Chao-1, and Shannon indices, were observed under different tree ages for **(A–C)** red soil and **(D–F**) paddy soil. Here, Age_10, Age_20, and Age_30 denote the different pomelo tree ages of 10, 20, and 30 years, respectively.

Alpha diversity indexes evaluated bacterial richness and diversity, such as observed OTU number, Chao1, and Shannon. In red soil, bacterial species richness indices, such as the observed number of OTUs (Figure 3A) and Chao1 (Figure 3B), decreased with increasing tree age. The bacterial diversity, that is, Shannon diversity index, also decreased with increasing tree age (Figure 3C). Similarly, the bacterial richness and diversity also decreased with increasing tree age under paddy soil (Figures 3D–F). It suggests that younger trees (i.e., 10 and 20 years) harbor higher bacterial richness and diversity.

### Changes in bacterial communities with different tree ages in red and paddy soils

**Figure 4:**
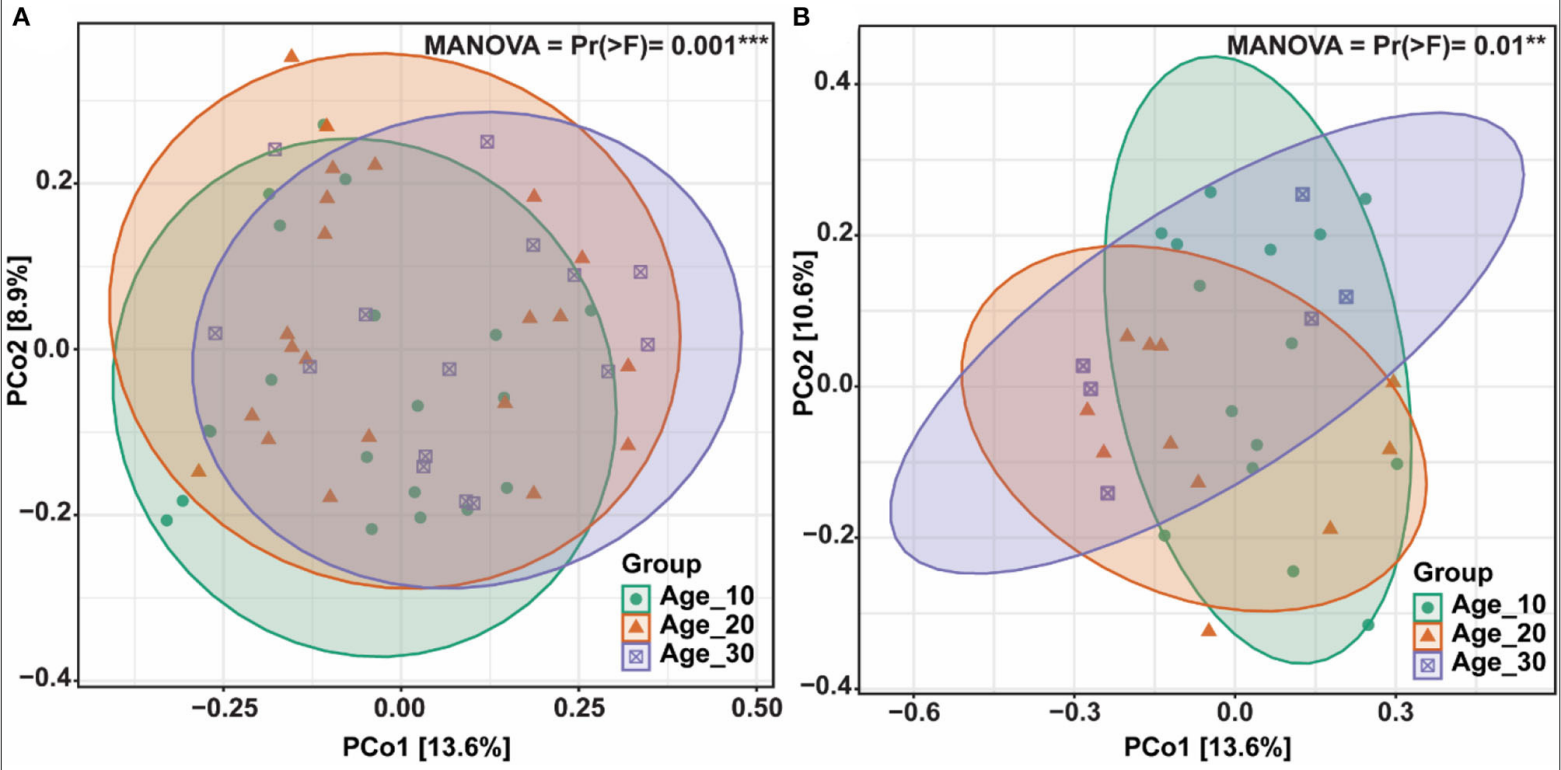
Bacterial community structures and composition. Differences in bacterial community composition under different tree ages were assessed by principal coordinate analysis (PCoA); **(A)** red soil; and **(B)** paddy soil. Here, Age_10, Age_20, and Age_30 denote the different pomelo tree ages of 10, 20, and 30 years, respectively.

The principal coordinate analysis (PCoA) was performed to evaluate the bacterial community composition across different tree ages under red and paddy soils. PCoA results showed that bacterial community under different tree ages was different from each other in red soil, where PCo1 and PCo2 accounted for variations of 13.6 and 8.9%, respectively. PERMANOVA also suggested that overall bacterial communities under different trees was highly significantly different (Pr(>*F*) = 0.001) (Figure 4A). Furthermore, PERMANOVA revealed significant bacterial community differences for Age_10 vs. Age_20 (*P* = 0.029), and Age_10 vs. Age_30 (*P* = 0.002), while non-significant differences were recorded for Age_20 vs. Age_30 (Table 1). Similarly, under paddy soil, the bacterial community was different under different tree ages, where PCo1 and PCo2 accounted for variations of 13.6 and 10.6%, respectively, and PERMANOVA analysis also showed significant differences (Pr(>*F*) = 0.01) (Figure 4B). Furthermore, PERMANOVA revealed significant bacterial community differences only for Age_10 vs. Age_30 (*P* = 0.016), while non-significant differences were recorded for Age_10 vs. Age_20 and Age_20 vs. Age_30 (Table 1). These results showed that tree age significantly affected bacterial community composition for red and paddy soils.

**Table 1:** Significance test of differences among fungal communities in different treatments using permutational analysis of variance based on Bray–Curtis distance.

**Soil type**	**Groups**	**Measure**	**Permutations**	*R* ^2^	***P*** **value**	**Significance**
Red soil	Age_10 vs. Age_20	Bray	999	0.038	0.029	^*^
	Age_10 vs. Age_30	Bray	999	0.06	0.002	^**^
	Age_20 vs. Age_30	Bray	999	0.034	0.157	
Paddy soil	Age_10 vs. Age_20	Bray	999	0.056	0.06	
	Age_10 vs. Age_30	Bray	999	0.081	0.016	^*^
	Age_20 vs. Age_30	Bray	999	0.893	0.071	

Star represents significance (*, P ≤ 0.05; **, P ≤ 0.01; while no star represents non-significance).

### Soil physicochemical properties correlation with the bacterial community with different tree ages

**Figure 5:**
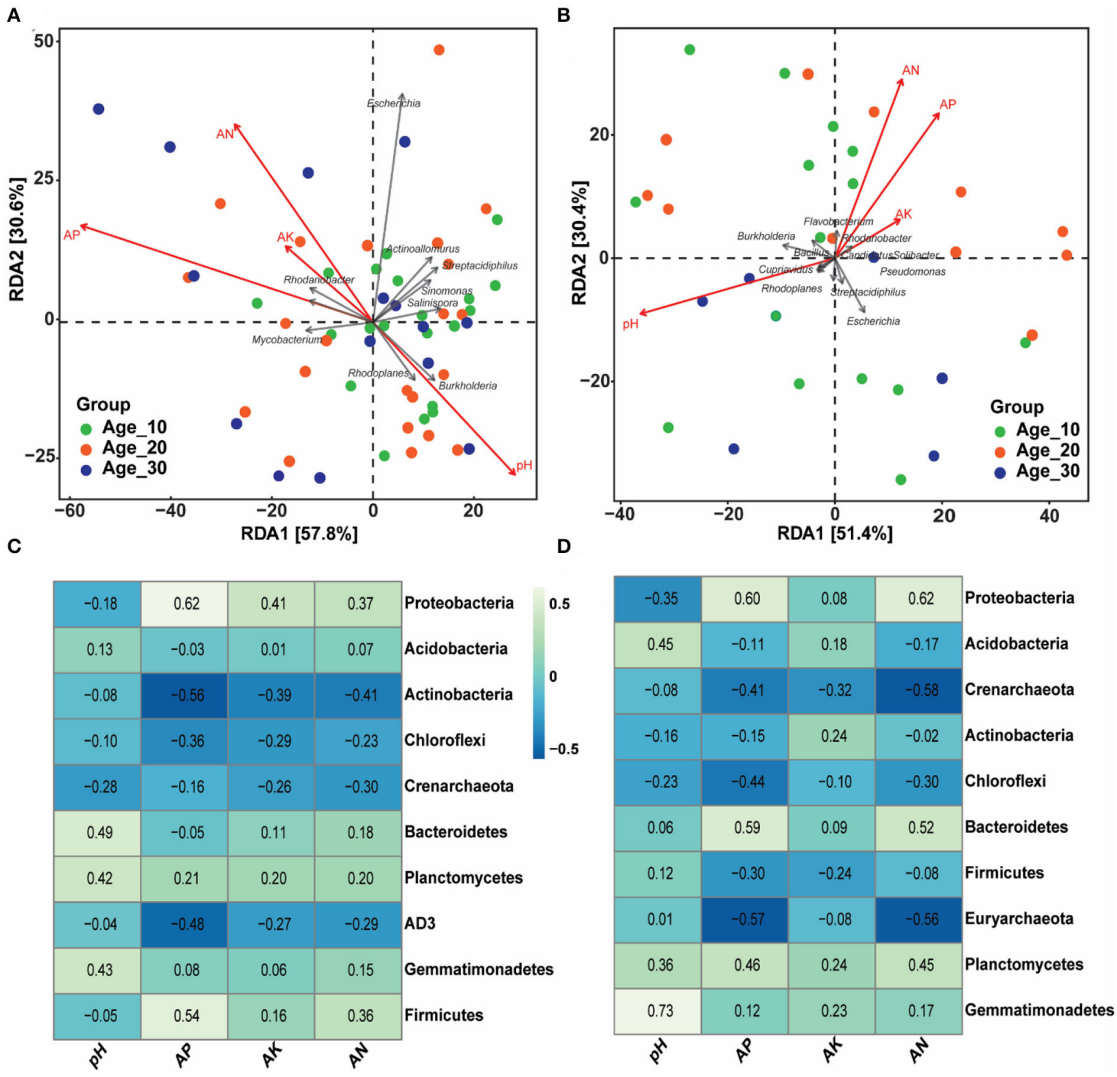
Influence of soil physicochemical characteristics on bacterial community. **(A,B)** Community–environment relationship using the RDA ordination plot of bacterial communities in red and paddy soils, respectively. **(C,D)** Heatmap showing the strength of correlation between the soil properties and bacterial community for red and paddy soils, respectively. Here, Age_10, Age_20, and Age_30 denote the different pomelo tree ages of 10, 20, and 30 years, respectively.

The effects of environmental factors, including soil pH, AP, AK, and AN, were examined on the bacterial population under red and paddy soils (Figure 5). RDA analysis showed that environmental factors, including soil pH, AP, and AN, were the most critical factors controlling the bacterial community under red soil (Figure 5A, Table 2). Similarly, in paddy soil, environmental factors such as soil pH and AP showed an empirical role in driving the bacterial community under the paddy soil (Figure 5B, Table 2). Moreover, at the phylum level, *Actinobacteria, Chloroflexi*, and *AD3* were negatively correlated, while Planctomycetes and Gemmatimonadetes showed a positive correlation with soil pH and AP, AK, and AN in red soil. In addition, *Proteobacteria* and *Firmicutes* were negatively correlated with soil pH, while positively correlated with other environmental factors in red soil (Figure 5C). *Proteobacteria, Actinobacteria*, and *Chloroflexi* were negatively associated with pH in the paddy soil. By contrast, *Acidobacteria, Bacteroidetes, Firmicutes, Planctomycetes*, and *Gemmatimonadetes* showed a positive correlation with soil pH (Figure 5D).

**Table 2:** Pearson correlation between the Bray–Curtis dissimilarity score and soil characteristics using the Mantel test.

	**Variable name**	**Corr-method**	**Corr_res**	**p_res**	**Significance**
Red soil	pH	Pearson	0.369	0.001	^***^
	AP (Available Phosphorous)	Pearson	0.203	0.002	^**^
	AK (Available Potassium)	Pearson	0.001	0.437	
	AN (Available Nitrogen)	Pearson	0.131	0.033	^*^
Paddy soil	pH	Pearson	0.251	0.003	^**^
	AP (Available Phosphorous)	Pearson	0.233	0.009	^**^
	AK (Available Potassium)	Pearson	−0.048	0.676	
	AN (Available Nitrogen)	Pearson	0.073	0.215	

Star represents significance (*, P ≤ 0.05; **, P ≤ 0.01; ***, P ≤ 0.001, while no star shows non-significance).

**Figure 6:**
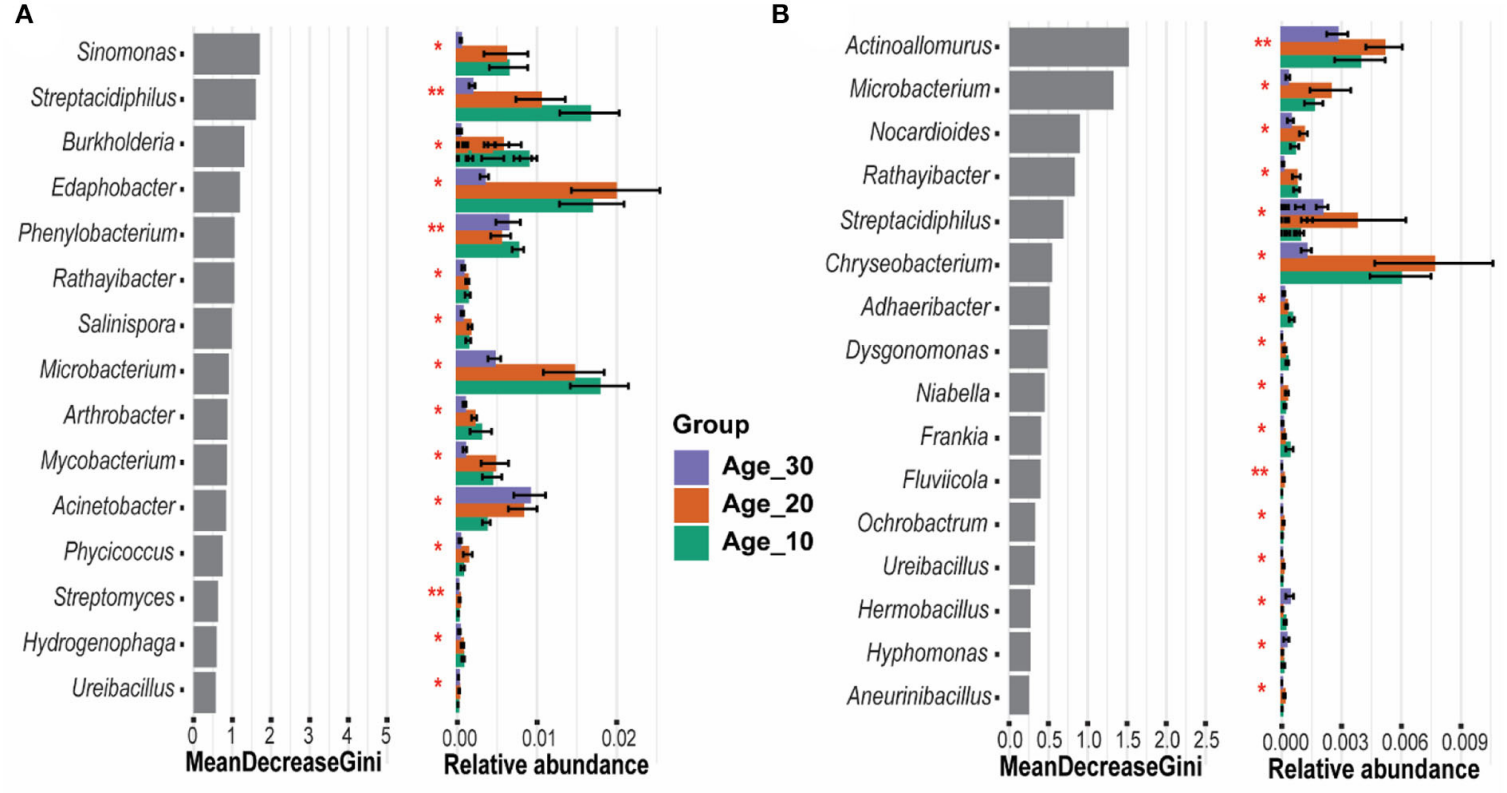
Random forest classification for evaluation of important genera. Gini index was used to determine the most significant genera, which exhibited the significance of each genus in distinguishing different age groups of **(A)** red soil and **(B)** paddy soil. Here, Age_10, Age_20, and Age_30 denote the different pomelo tree ages of 10, 20, and 30 years, respectively.

**Figure 7:**
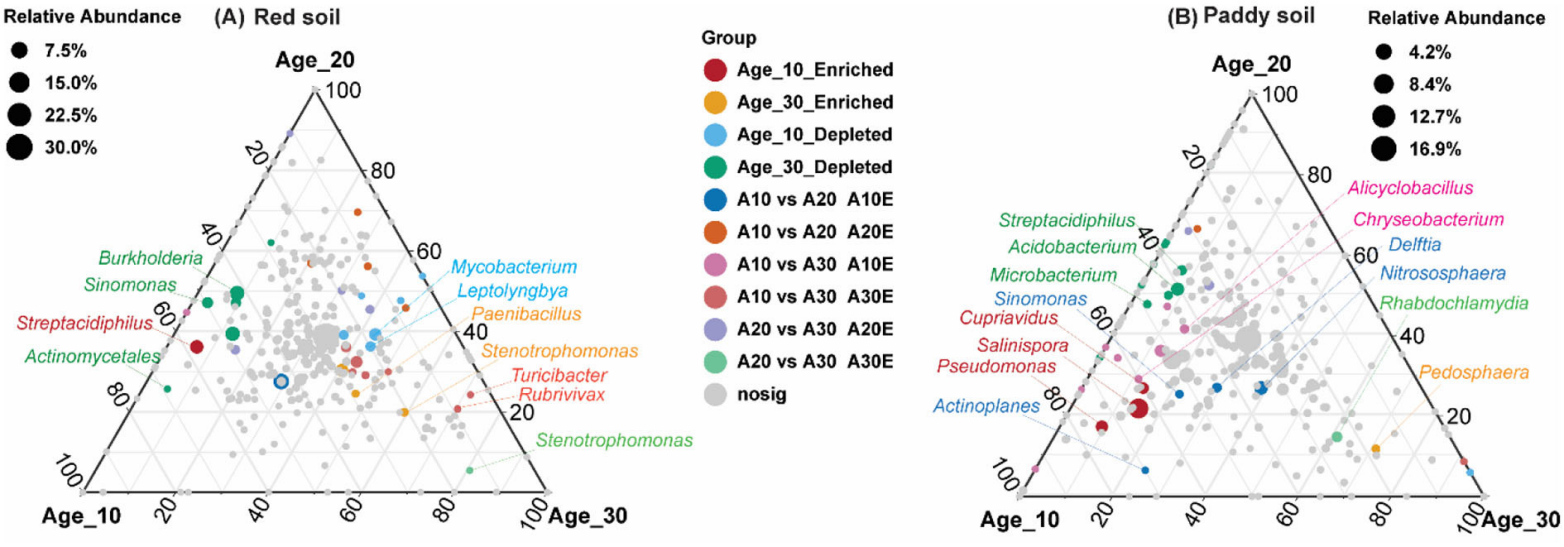
Ternary plot analysis. Ternary plot represents the soil bacterial communities with significant differences in relative abundance in different tree ages at the genus level in **(A)** red soil and **(B)** paddy soil. Here, Age_10, Age_20, and Age_30 denote the different pomelo tree ages of 10, 20, and 30 years, respectively. Each point represents species, and the size of the point corresponds the relative abundance of each species. “Enriched” denotes the significant increase in relative abundance compared with others two, while “depleted” represents the significant lower relative abundance of a group than other two groups, and non-sig represents the non-significant differences among different age groups.

The random forest model determined the most important genera that classified the samples into different age groups. Results revealed that *Sinomonas, Streptacidiphilus, Burkholderia, Edaphobacter*, and *Phenylobacterium* were the most important genera under red soil and found significant differences in their abundances under different age groups (Figure 6A). In the case of paddy soil, the most important genera were *Actinoallomurus, Microbacterium, Nocardioides, Rathayibacter*, and *Streptacidiphilus*, and their abundances were significantly affected among different tree age groups (Figure 6B). Moreover, ternary plot analysis was performed to identify the specific enriched and depleted bacterial genera under different tree ages. The results showed that the pomelo tree of Age_10 in red soil was significantly enriched with *Streptacidiphilus*, while *Mycobacterium* and *Leptolyngbya* were depleted significantly. Under Age_20, we did not observe any significant enriched or depleted genera. By contrast, the pomelo tree of Age_30 was significantly enriched with *Rhodanobacter, Paenibacillus*, and *Stenotrophomonas*, while depleted with *Burkholderia, Sinomonas*, and *Actinomycetales*. Overall, genera enrichment for Age_30 was higher than that for Age_10 and Age_20 (Figure 7A). For paddy soil, *Cupriavidus, Pseudomonas*, and *Salinispora* were significantly enriched under Age_10, while *Pedosphaera* was enriched under Age_30. Hence, generally, genera enrichment decreased with increasing tree age under paddy soil (Figure 7B).

### Co-occurrence network analysis

**Figure 8:**
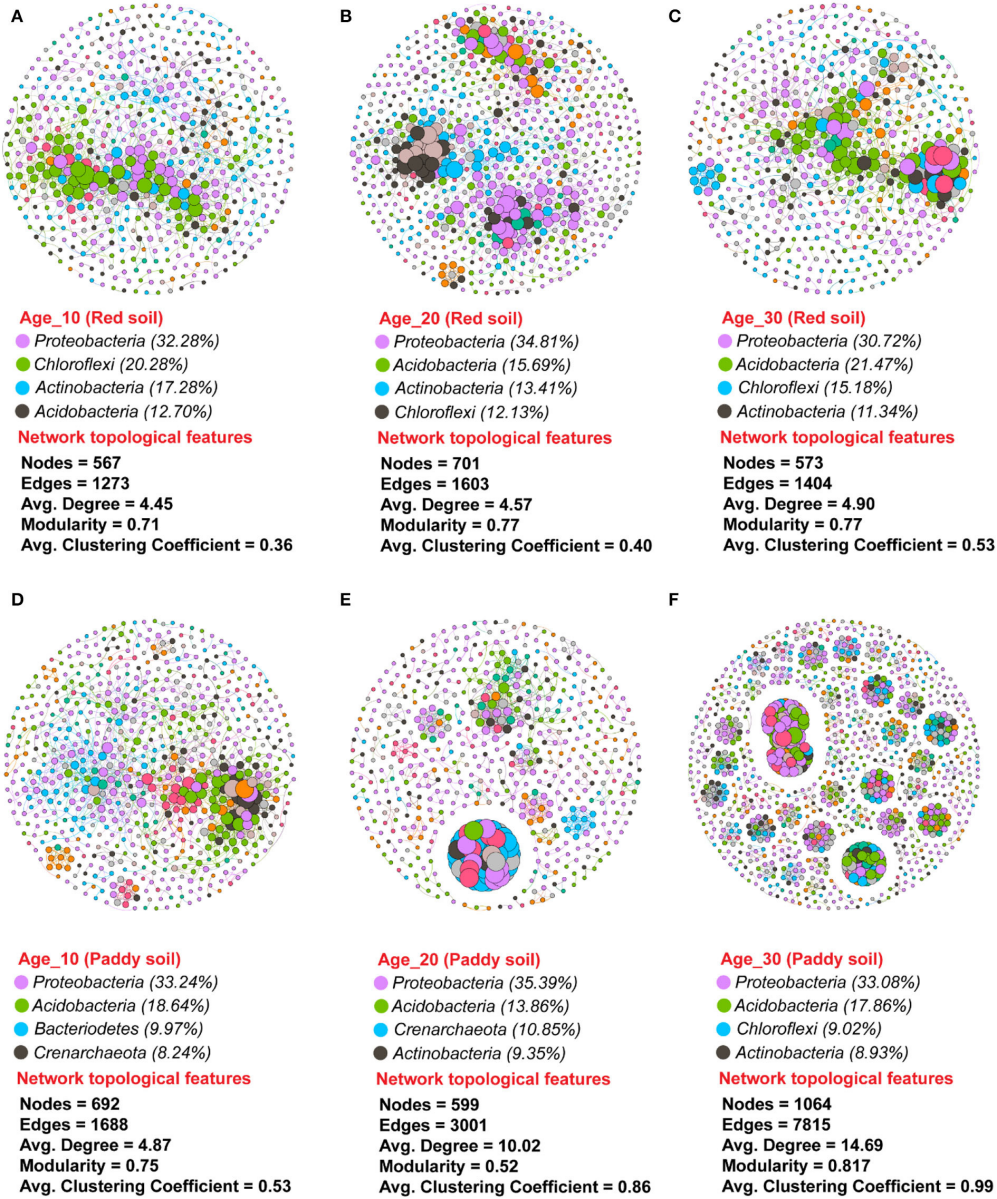
Dynamic changes in bacterial co-occurrence networks under different groups of tree ages. Co-occurrence network was investigated for **(A–C)** red soil and **(D–F)** paddy soil. Each node in the network has a different color based on its phylum. The size of each node indicates the taxa's relative abundance. The connections are significant (*P* ≤ 0.05) and strongly correlated (Spearman's ρ ≥ 0.6). Here, Age_10, Age_20, and Age_30 denote the different pomelo tree ages of 10, 20, and 30 years, respectively.

A non-random co-occurrence network pattern of phylotypes was studied to investigate how the interactions between phylotypes respond to variations in tree ages under red and paddy soils. The values of the modularity index ranged from 0.71 to 0.77 under red soil, while 0.52–0.81 under paddy soil (Figure 8). The modularity index value greater than the proposed threshold value, that is, 0.40, indicated that all networks were modularly structured. The number of nodes increased with increasing tree age in red soil, and the lowest number of nodes was found in Age_10. However, the number of edges, that is, 1,603 and 1,404; the average degree (the tendency of nodes to cluster together), that is, 4.57 and 4.90; and the average clustering coefficient under (degree of nodes connectivity), that is, 0.40 and 0.53 under red soil in Age_20 and Age_30, respectively, were higher than Age_10 edges (1,273), average degree (4.45), and average clustering coefficient (0.36). The bacterial species *Proteobacteria, Acidobacteria, Actinobacteria*, and *Chloroflexi* represented the most dominant bacterial community in red soil (Figures 8A–C). Similarly, under the paddy soil, the number of nodes increased with increasing tree, and the highest nodes were recorded under Age_30. Moreover, the number of edges (1,064), average degree (14.69), and average clustering coefficient (0.99) under Age_30 were also higher than those under Age_10 and Age_20. Similar to red soil, in the paddy soil, the bacterial species *Proteobacteria, Acidobacteria, Actinobacteria, Chloroflexi*, and *Bacteroidetes* were the most dominant bacterial community (Figures 8D–F). However, these findings revealed that bacterial species tended to interact more closely and formed more complex network structures with increasing tree age, for example, Age_20 and Age_30 compared to Age_10 tree.

## Discussion

Soil microbes are the key components of soil ecosystems and play a significant role in maintaining a healthy and stable environment for tree growth and development. In this study, we examined the changes in soil physicochemical properties, bacterial diversity, and community structure under different tree ages in the red and paddy soils. The soil properties, bacterial diversity, and community structure differed significantly among pomelo trees of different ages in red and paddy soils.

Soil physicochemical properties are an important index to evaluate the soil quality that ultimately affects the soil fertility and microbial community (Shao et al., 2020; Yin et al., 2021). Various studies have indicated that tree age substantially impacts soil physicochemical properties (Sharma et al., 2009; Yin et al., 2021). For instance, soil pH is one of the most critical determinants of soil quality. At present, contrasting results have been reported on the effects of tree age on soil pH (Figure 1). Some studies have found that soil pH is increased significantly with an increase in tree age (Malchair and Carnol, 2009), while others reported that the pH value of the soil steadily drops as the tree age increases (Bormann and DeBell, 1981; Sharma et al., 2009), indicating a trend of soil acidification. In this experiment, the pH value was significantly decreased with increasing tree age in red soil, consistent with previous findings (Bormann and DeBell, 1981; Sharma et al., 2009). The possible reason could be the reduced base saturation caused by H^+^ production in the nitrification process, with H^+^ substituting the base cations leached from the soil with ${\text{NO}}_{3}^{-}$ (Binkley and Sollins, 1990). Additionally, leaching of base cations with water, organic matter accumulation, atmospheric acid deposition, and soil microbial respiration may all contribute to a reduction in soil pH (Hinsinger and Jaillard, 1993; Wong et al., 2008). While, in the paddy soil, there was no significant difference in pH between different tree ages, the availability of soil nutrients was significantly affected, and the same results have been reported in a previous study (Yin et al., 2021). The concentration of phosphorous availability (AP) was increased with a decrease in soil pH because it has been reported that a decrease in pH may result from increasing the activity of proton-coupled solute transporters and enhancing the anion uptake (White, 2011; da Silva Cerozi and Fitzsimmons, 2016; Yin et al., 2021). The availability of potassium (AK) and nitrogen (AN) increased in red soil while decreasing with increasing tree age in paddy soil. The soil pH significantly affects nutrient availability (Zhao et al., 2011). Most plant nutrients are available in soil that is slightly acidic to slightly alkaline. Various plant nutrients are unavailable in highly acidic or alkaline soils owing to chemical reactions in the soil that fix nutrients and transform them into a form that is unavailable to plants. Hence, tree ages have a substantial impact on soil physicochemical properties.

The bacterial community in red and paddy was dominated by *Proteobacteria* and *Acidobacteria* (Figure 2), which is consistent with the previous studies on soil microbiome (Zhou et al., 2015; Wang et al., 2016). However, the relative abundance of *Proteobacteria* and *Acidobacteria* was increased in 20- and 30-year-old trees compared to 10-year-old trees in red soil. This is because *Proteobacteria* belongs to the copiotroph group, and its relative abundance increases with an increase in resources (Eilers et al., 2010), as in the present study, we found AP, AK, and AN increased with increasing tree age in red soil. By contrast, in paddy soil, *Proteobacteria* relative abundance decreased because of the nutrient-poor environment, for example, a decrease in AK and AN under Age_30. *Acidobacteria* relative abundance increased with tree age, and the highest relative abundance was found under Age_30 in red and paddy soils. This is because *Acidobacteria* favors the acidic environment because it was isolated from an acidic mineral environment (Kishimoto et al., 1991), and Jones et al. (2009) found that the relative abundance of *Acidobacteria* increased under low soil pH, consistent with our findings that relative abundance of *Acidobacteria* increased under low soil pH. These findings imply that soil pH plays a vital role in changing the relative abundance of *Acidobacteria* across the age sequence of the pomelo plantation.

Under both red and paddy soils of the present study, the bacterial richness and diversity index values decreased substantially with increasing tree age (Figure 3), which concurs with the results of previous studies showing a high level of diversity in 10- and 18-year-old trees, while diversity decreased in 30-year-old trees (Zhou et al., 2017), and some other studies also found that bacterial diversity decreased with increasing plantation age (Wu et al., 2015; Zhu et al., 2019). It has been widely accepted that tree development affects the soil microbiota through various pathways (Mitchell et al., 2012; Dang et al., 2017). For example, tree cover has a direct impact on the amount of light available as well as the composition of the understory, which affects the microbial population in the soil. As a result, differences in the tree canopy of different tree ages may indirectly impact soil bacterial diversity *via* understory litter substrate and root system (Dang et al., 2017). The other possible explanation for the decrease in bacterial richness and diversity could be the low soil pH because we found that with increasing tree age, soil pH was significantly decreased, and it has been found that soil acidification significantly decreases the bacterial diversity (Yun et al., 2016). These findings suggest that tree age significantly shifts the underlying soil bacterial communities, in line with the study of Wu et al. (2015), who found that plant age had a substantial effect on microbial communities in *Pinus elliottii*.

Changes in tree age significantly affect soil physicochemical properties, which in turn affect the soil bacterial community structure (Qu et al., 2020; Yin et al., 2021). For example, soil pH has been recognized as the most influential factor that drives the variation of soil bacterial community assembly (Siciliano et al., 2014; An et al., 2019). In the current study, we also found that soil pH, AP, and AN effects were significant on the bacterial community (Figure 5, Table 2) and consistent with various studies (Liu et al., 2014; An et al., 2019). This may explain the changes in the bacterial community under different tree ages. *Proteobacteria* was positively correlated with AP, AK, and AN because it is categorized into a copiotroph group that prefers high-nutrient soil resources (Eilers et al., 2010). *Acidobacteria* was positively correlated with soil pH and consistent with previous findings (Wang et al., 2018; Muneer et al., 2022). Various studies have reported inconsistent results of soil pH on *Acidobacteria*, where some studies also have shown a negative correlation (Jones et al., 2009; Navarrete et al., 2013). It is possible due to a better suitable environment for their adaptation. For example, it has been found that some *Acidobacteria* usually prefers high acidic conditions (i.e., pH ~ 2–3) compared to other environments (Kleinsteuber et al., 2008). Despite the substantial (positive or negative) association with pH, it is still unknown whether pH affects *Acidobacteria* directly or is dependent on other environmental factors that fluctuate with soil pH. Overall, we found positive and negative correlations between bacterial community and soil pH. The researchers have proposed two possible hypotheses explaining the relationship between soil pH and bacterial community. First, soil pH exerts a physiological restraint on the bacterial community, affecting the competitive outcomes or limiting the net growth of taxa that cannot live if the soil pH goes beyond a particular range. The majority of bacterial intracellular pH is close to neutral, and hence, the severe pH level may cause substantial pressure, and as a result, some taxa can withstand better than others (Lauber et al., 2009). Second, soil pH may not have a direct effect on the bacterial community, but rather serve as an integrating factor that offers a comprehensive assessment of soil environments. Various soil properties (e.g., nutrient availability, organic carbon, and soil moisture) are directly or indirectly related to soil pH, and these factors may attribute to changes in the bacterial community (Bissett et al., 2011; Zhou et al., 2017).

Random forest analysis showed that the most important genera in red soil belonged to phylum *Actinobacteria* (i.e., *Sinomonas* and *Streptacidiphilus*) and *Proteobacteria* (i.e., *Burkholderia* and *Phenylobacterium*) (Figure 6). *Sinomonas* has been reported to help in nitrogen fixation and plant growth (Lee et al., 2015; Susilowati et al., 2015), while *Streptacidiphilus* has an important role in biodegradation (e.g., chitin and lignocellulose), secondary metabolite production, and plant growth-promoting potential (Bentley et al., 2002). *Burkholderia* and *Phenylobacterium* are ubiquitous and found in a variety of soils owing to their functions in nitrogen fixation, degradation of organic compounds, and their interaction with plants, including beneficial and pathogenic (Compant et al., 2008). Likewise, another important genus, *Edaphobacter*, belonging to the phylum *Acidobacteria*, has been found mostly in acidic and calcareous soils and could play a key role in the regulation of nutrient cycling and plant growth promotion (Kalam et al., 2020). Similarly, in paddy soil, the most important identified taxa, including *Actinoallomurus, Microbacterium, Nocardioides, Rathayibacter*, and *Streptacidiphilus*, belonged to *Actinobacteria*. These have several important functions, including cycling of carbon, nitrogen, phosphorous, and several other elements in soil, and decomposition of organic matter (Holmalahti et al., 1994; Zhang et al., 2019). According to the results of ternary plot analysis (Figure 7), under red soil, the relative abundance of specific genera, including *Rhodanobacter* and *Paenibacillus*, significantly increased with increasing tree age, and these bacterial communities play an active role in N-cycling (Liu et al., 2019; Peng et al., 2022), while *Stenotrophomonas* was found to contribute to N and S cycles and could play a substantial role in improving plant growth (Ryan et al., 2009). Likewise, in paddy soil, the N cycling-related bacterial community of *Cupriavidus* and *Pseudomonas* (Mirza et al., 2006; Wang et al., 2020) was significantly higher under Age_10, and the specific genera enrichment decreased with increasing tree age. However, these findings highlight the important taxa in different soils of pomelo orchard and provide a reference that could be useful for understanding their ecological significance under global changes.

In general, soil microorganisms do not function as individuals, but instead establish complex interaction networks. As a result, co-occurrence network analysis is an effective method for determining how related soil microbiota interact with environmental changes. The co-occurrence networks revealed that the majority of nodes belonged to *Proteobacteria, Acidobacteria, Actinobacteria*, and *Chloroflexi* across all tree ages (Figure 8). This finding was consistent with that of a previous study (Yan L. et al., 2021), further demonstrating that *Proteobacteria, Acidobacteria, Actinobacteria*, and *Chloroflexi* are the most dominant phyla in pomelo orchards of red and paddy soils. The highly connected nodes revealed strong bacterial association under 20 and 30 years old trees compared to 10 years old in red and paddy soils, and these taxa are known as keystone taxa (Guo et al., 2019). These keystone taxa have prime importance for sustaining the co-occurrence network structure (Faust and Raes, 2012). As a result of the complex network connectivity, the bacterial community under 20- and 30-year-old trees could be more tolerant to environmental changes as it is generally documented that bacterial communities with higher interconnectivity are more resistant to environmental changes than simple networks with less connectivity (Santolini and Barabási, 2018).

Note that in this study, we only focused on how the dynamic changes in tree age could affect the soil bacterial diversity, community composition, and functioning. However, the impact of root exudates on the soil microbial population was not considered owing to the key focus of tree age. Indeed, the root exudates affect the microbial diversity and community composition. Hence, further studies are needed to understand the composition of the exudate in the orchard system and to better link the tree age, the root exudate, and soil microbiota.

## Conclusion

This study revealed that tree age has a significant effect on changing the soil physicochemical properties, and contrasting patterns of changes in soil physicochemical properties were recorded under different tree ages in the red and paddy soils. Moreover, tree age also substantially affected soil bacterial diversity and community composition. Bacterial richness and diversity decreased with increasing tree age in both soil types, while tree age formed the distinct bacterial community structures under red and paddy soils. Soil pH, AP, and AN in red soil, while pH and AP in paddy soil were the key environmental variable contributing to the shift in the bacterial community structure. However, the co-occurrence network revealed that bacterial species formed a complex network structure with increasing tree age, indicating a more stable microbial association under 20- and 30-year-old than 10-year-old pomelo trees. Moreover, the random forest model identified the most important genera in red soil, for example, *Sinomonas* and *Streptacidiphilus*, which play a key role in nitrogen fixation, plant growth, biodegradation, etc., while paddy soil was dominated by *Actinoallomurus Microbacterium*, etc., which have key functions including cycling of carbon, nitrogen, phosphorous, and several other elements in soil, and decomposition of organic matter. These findings provide valuable information regarding the importance of microbes for the sustainable management of pomelo orchards by optimizing fertilizer input for different ages of trees.

## Data availability statement

The datasets presented in this study can be found in online repositories. The names of the repository/repositories and accession number(s) can be found below: https://www.ncbi.nlm.nih.gov/, PRJNA779204.

## Author contributions

CZ: conceptualization and writing—original draft. KK and YZ: data curation and software. WY and LW: investigation, data curation, and conceptualization. MZM and BJ: writing—review and editing. MAM: writing—original draft, software, visualization, and writing—review and editing. All authors contributed to the manuscript and approved the submitted version.

## Funding

This research was funded by the National Natural Science Foundation of China (41601244), the Open Research Foundation of International Magnesium Institute (IMI2018-09), and the Science and Technology Innovation Foundation of FAFU (CXZX2020076A).

## Conflict of interest

The authors declare that the research was conducted in the absence of any commercial or financial relationships that could be construed as a potential conflict of interest.

## Publisher's note

All claims expressed in this article are solely those of the authors and do not necessarily represent those of their affiliated organizations, or those of the publisher, the editors and the reviewers. Any product that may be evaluated in this article, or claim that may be made by its manufacturer, is not guaranteed or endorsed by the publisher.
